# Changes in Buprenorphine Prescribing in Community Health Centers

**DOI:** 10.1001/jamahealthforum.2024.0634

**Published:** 2024-04-26

**Authors:** Daniel M. Hartung, Robert W. Voss, Steffani R. Bailey, Nathalie Huguet, John Muench

**Affiliations:** 1 College of Pharmacy, Oregon State University Portland; 2 OCHIN, Inc, Portland, Oregon; 3 Department of Family Medicine, Oregon Health & Science University, Portland

## Abstract

This cohort study characterizes changes in buprenorphine prescribing among community health center clinicians between 2016 and 2021.

## Introduction

Over the last decade, the opioid crisis has worsened, particularly among underserved patients with economic, geographic, or cultural barriers to care.^1^ Community health centers (CHCs) provide addiction treatment services in these communities, regardless of patients’ ability to pay.^2^ In 2019, CHCs reported increased numbers of patients treated for opioid use disorder (OUD), mostly with buprenorphine.^2^ Using data from the OCHIN Inc network, we characterized changes in buprenorphine prescribing among CHC clinicians between 2016 and 2021. We examined changes by rural vs urban location and physicians vs nonphysician clinicians (nurse practitioners [NPs] and physician assistants [PA]) who gained buprenorphine prescriptive authority in 2017.^3^

## Methods

This cohort study was approved by the Oregon Health & Science University Institution Review Board, which waived the need for informed consent. The study followed the STROBE reporting guideline.

Data were obtained from the OCHIN Inc network of CHCs, which all operate on a shared electronic health record. We measured the proportion of clinicians providing at least 1 buprenorphine-containing medication order, excluding pain-only formulations, at 166 clinics active in both 2016 and 2021 who had at least 12 visits in that year. Multivariable logistic regressions estimated changes between 2021 and 2016 in the proportion of clinicians prescribing buprenorphine overall, by clinician type, and by rural vs urban location.^4^ We reported adjusted odds ratios (AORs) using interaction of year with clinician type and location to evaluate whether changes differed across each stratum. The triple interaction among period, clinician, and location assessed whether changes across clinician type differed by clinic location. We used SAS, version 9.4, for statistical analysis.

## Results

A total of 2186 unique clinicians contributed to one or both years of the study (69.9% female) for 978 275 patients present in one or both years (58.2% female). While the patient comorbidity burden increased, the number and characteristics of CHC clinicians and patients in the study sample remained relatively consistent from 2016 to 2021 (Table 1). The percentage of CHC patients receiving buprenorphine increased from 0.5% to 1.4%.

**Table 1.** OCHIN Community Health Center Clinician and Patient Characteristics, 2016 and 2021^a^

Characteristic	2016	2021
Clinicians^b^	1383 (100)	1334 (100)
Location^c^		
Urban	1142 (82.6)	1074 (80.5)
Rural	241 (17.4)	260 (19.5)
Type		
Physician	773 (55.9)	680 (51.0)
NP or PA	610 (44.1)	654 (49.0)
Patients	603 406 (100)	572 165 (100)
OUD	7415 (1.2)	9429 (1.6)
Buprenorphine prescription	2955 (0.5)	8193 (1.4)
Sex		
Women	361 361 (59.9)	335 802 (58.7)
Men	242 045 (40.1)	236 363 (41.3)
Race and ethnicity		
Hispanic	169 298 (28.1)	175 108 (30.6)
Non-Hispanic American Indian or Alaska Native	3317 (0.5)	3096 (0.5)
Non-Hispanic Asian	27 137 (4.5)	25 097 (4.4)
Non-Hispanic Black	107 246 (17.8)	101 101 (17.7)
Non-Hispanic Native Hawaiian or Other Pacific Islander	2135 (0.4)	1129 (0.2)
Non-Hispanic White	264 077 (43.8)	230 175 (40.2)
Non-Hispanic multiple races	4706 (0.8)	5213 (0.9)
Unknown	25 490 (4.2)	31 246 (5.5)
Age, y		
18-24	78 819 (13.1)	69 636 (12.1)
25-34	120 317 (19.9)	110 995 (19.4)
35-44	107 913 (17.9)	107 022 (18.7)
45-54	114 787 (19.0)	101 065 (17.7)
55-64	106 925 (17.7)	101 146 (17.7)
≥65	74 645 (12.4)	82 301 (14.4%)
Household income		
≤138% of FPL	280 333 (46.5)	261 454 (45.7)
>138% of FPL	94 371 (15.6)	123 144 (21.5)
Missing	228 702 (37.9)	187 567 (32.8)
Payer		
Medicaid	290 460 (48.1)	248 308 (43.4)
Medicare	88 257 (14.6)	82 951 (14.5)
Other public insurance	27 807 (4.6)	17 579 (3.1)
Private	101 065 (16.7)	118 603 (20.7)
Uninsured	95 817 (15.9)	94 732 (16.6)
Missing	0	9992 (1.7)
Mental health conditions		
0	538 690 (89.3)	429 502 (75.1)
1	41 085 (6.8)	80 444 (14.1)
≥2	23 631 (3.9)	62 219 (10.9)
Somatic health conditions		
0	460 184 (76.3)	317 143 (55.4)
1	93 828 (15.5)	131 386 (23.0)
2-3	42 858 (7.1)	101 049 (17.7)
≥4+	6536 (1.1)	22 587 (3.9)

Abbreviations: FPL, federal poverty level; NP, nurse practitioner; OUD, opioid use disorder; PA, physician assistant.

^a^ Data are presented as the No. (%) of participants.

^b^ Sample includes clinicians with at least 12 visits during year in 166 clinics active in both periods.

^c^ Designation from zip code level Rural Urban Classification Code.^4^

Buprenorphine prescribing increased from 8.9% to 37.5% of modeled clinicians (AOR, 6.12 [95% CI, 4.78-7.83]) (Table 2). Buprenorphine prescribing increased similarly in rural (AOR, 4.36 [95% CI, 2.73-7.05]) and urban (AOR, 6.86 [95% CI, 5.15-9.14]) clinics. Prescribing increased significantly for nonphysicians (AOR, 10.69 [95% CI, 6.81-16.8]) and physicians (AOR, 4.66 [95% CI, 3.46-6.25]), with a greater increase for nonphysicians (AOR, 2.30 [95% CI, 1.35-3.91]).

**Table 2.** Modeled Proportions and Multivariable Logistic Regression of Buprenorphine-Prescribing Clinicians^a^

Buprenorphine-prescribing clinicians	Modeled proportion, %		AOR (95% CI)
	2016	2021	
Overall	8.9	37.5	6.12 (4.78-7.83)
Clinician type			
Physician	10.4	35.0	4.66 (3.46-6.25)
NP or PA	2.2	19.6	10.69 (6.81-16.8)
Clinician type × period interaction	NA	NA	2.30 (1.35-3.91)
Clinic location			
Urban	8.3	38.3	6.86 (5.15-9.14)
Rural	20.3	52.7	4.36 (2.73-7.05)
Clinic location × period interaction	NA	NA	0.64 (0.37-1.11)
Clinician type by clinic location			
Urban physician	10.2	35.8	4.92 (3.54-6.86)
Urban NP or PA	1.3	20.5	18.92 (9.79-36.55)
Clinician type × period in urban location	NA	NA	3.84 (1.86-7.95)
Rural physician	20.4	49.0	3.75 (1.93-7.28)
Rural NP or PA	8.2	30.5	4.93 (2.52-9.64)
Clinician type × period in rural location	NA	NA	1.32 (0.52-3.35)
Clinician type × period × clinic location	NA	NA	0.34 (0.11-1.12)

Abbreviations: AOR, adjusted odds ratio; NA, not applicable; NP, nurse practitioner; PA, physician assistant.

^a^ Sample includes clinicians with at least 12 visits during the year in 166 clinics active in both periods. Proportions and AORs were estimated from multivariable logistic regressions. Interaction terms reflect change in buprenorphine prescribing from 2016 to 2021 by clinician type (NP or PA vs physician [reference]) or by clinic location (rural and urban designation from zip code level Rural Urban Classification Code^4^).

The increase in buprenorphine prescribing for nonphysicians was pronounced in urban clinics, where the AOR for buprenorphine prescribing (AOR, 18.92 [95% CI, 9.79-36.55]) was significantly higher than the increase observed for physicians (AOR, 4.92 [95% CI, 3.54-6.86]) (Table 2). In rural clinics, buprenorphine prescribing increases were similar between physicians and nonphysicians; however, the triple interaction estimate was not significant, indicating differences in changes by clinician type did not vary by location.

## Discussion

From 2016 to 2021, the odds of prescribing buprenorphine among clinicians in the OCHIN Inc CHC network increased significantly (AOR, 6.12 [95% CI, 4.78-7.83]); more than one-third of CHC clinicians prescribed buprenorphine in 2021. The increase was largest for NPs and PAs, particularly in urban clinics. However, physicians remained the most likely to prescribe buprenorphine in 2021, with about half prescribing buprenorphine in rural clinics.

Federal efforts to expand treatment access for OUD, culminating in the elimination of the Drug Enforcement Agency X-waiver in 2023, have resulted in more clinicians with buprenorphine prescriptive authority.^5^ Prior to its removal, only 6% of physicians, 9% of NPs, and 5% of PAs nationwide had an X-waiver.^6^ With over one-third of all clinicians issuing buprenorphine prescriptions, buprenorphine prescribing in the CHCs substantially exceeds national estimates.

Study limitations include our inability to verify whether clinicians had obtained an X-waiver or whether patients were being treated for OUD. External validity may also be limited because our sample was disproportionately represented by clinics in western states.

Community health centers play a critical role in providing care to underserved communities disproportionally affected by the opioid crisis. With elimination of the X-waiver, CHCs are well positioned to expand life-saving buprenorphine access to this vulnerable and high-risk population.

## Notes

Supplement.
Data Sharing Statement
