# Supplementary material for: Changes in Buprenorphine Prescribing in Community Health Centers
Source: JAMA Health Forum. 2024 Apr 26;5(4):e240634. doi: 10.1001/jamahealthforum.2024.0634 (PMC11065159; doi:10.1001/jamahealthforum.2024.0634)
Supplement: Supplement. — Data Sharing Statement [file jamahealthforum-e240634-s001.pdf]

# Data Sharing Statement

Hartung. Changes in Buprenorphine Prescribing in Community Health Centers. *JAMA Health Forum*. Published April 26, 2024. doi:10.1001/jamahealthforum.2024.0634

**Data**
**Data available:** No
